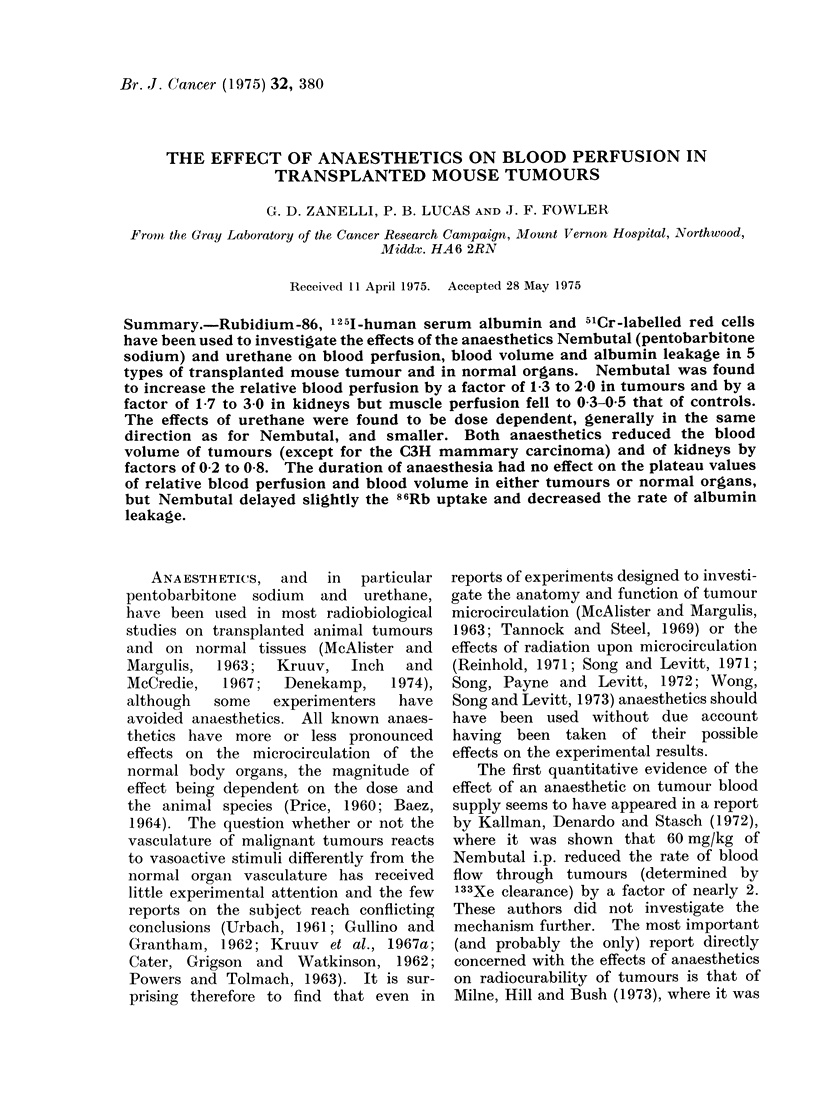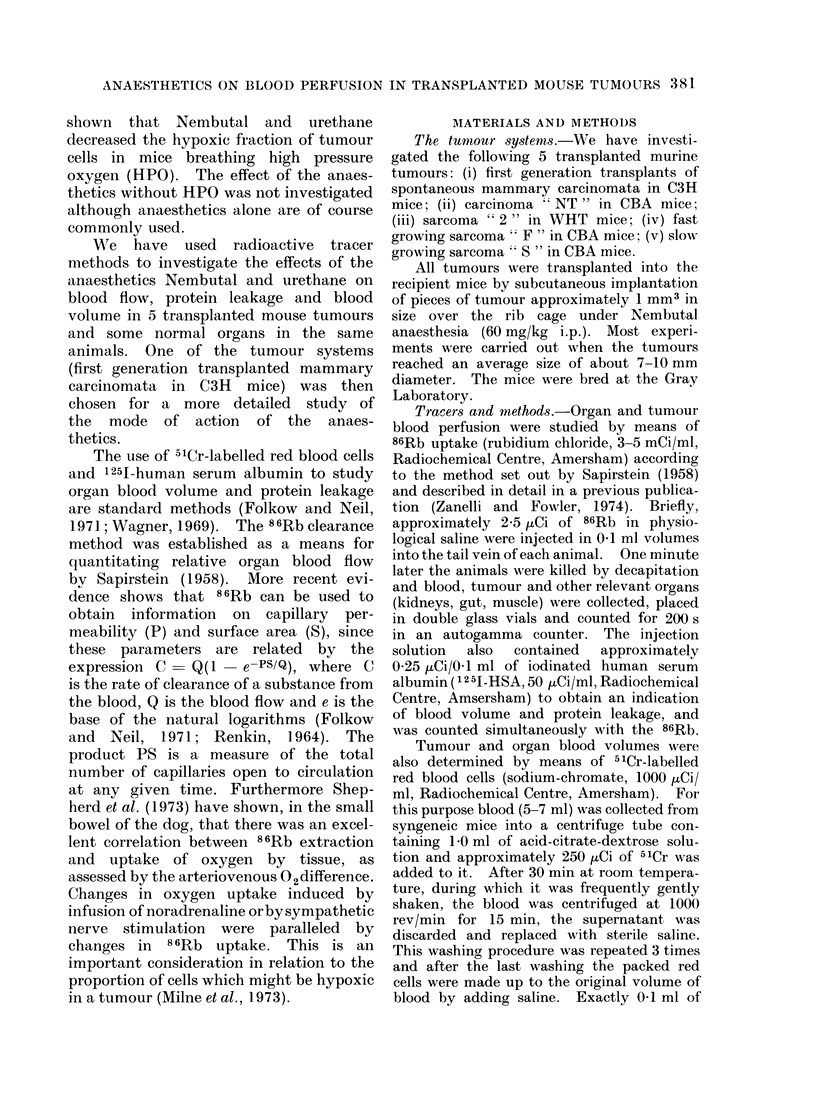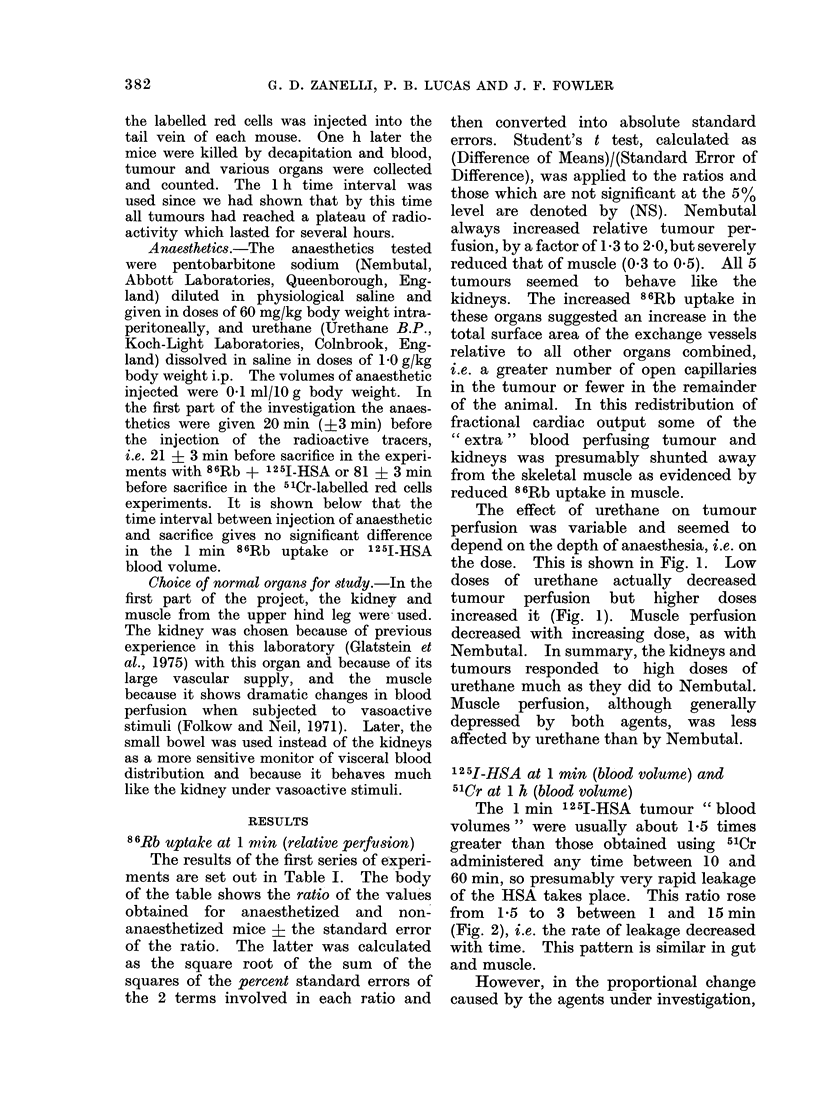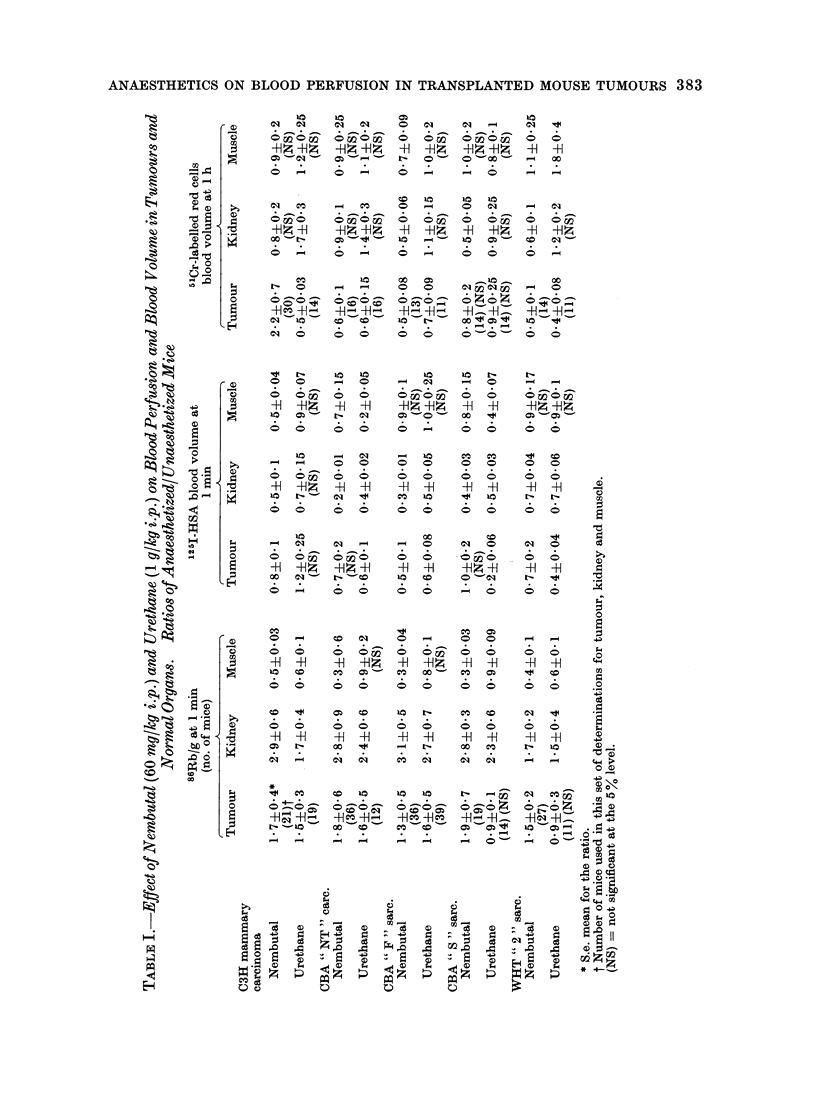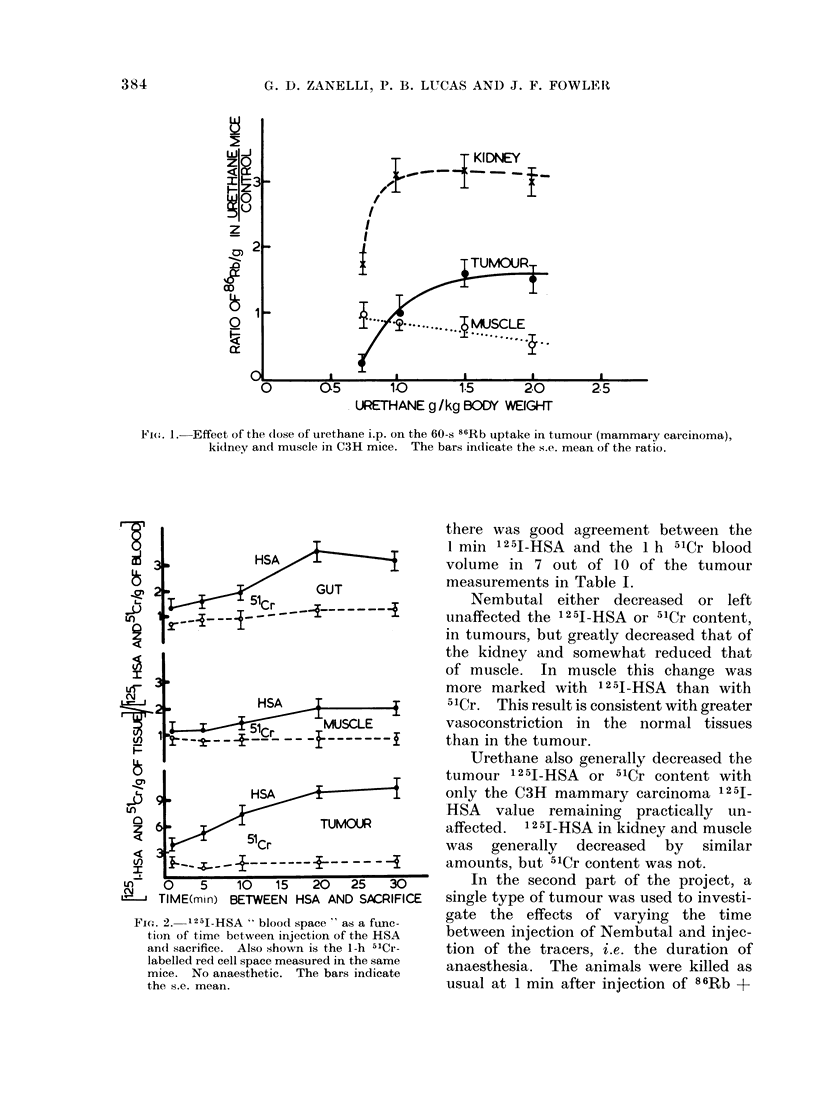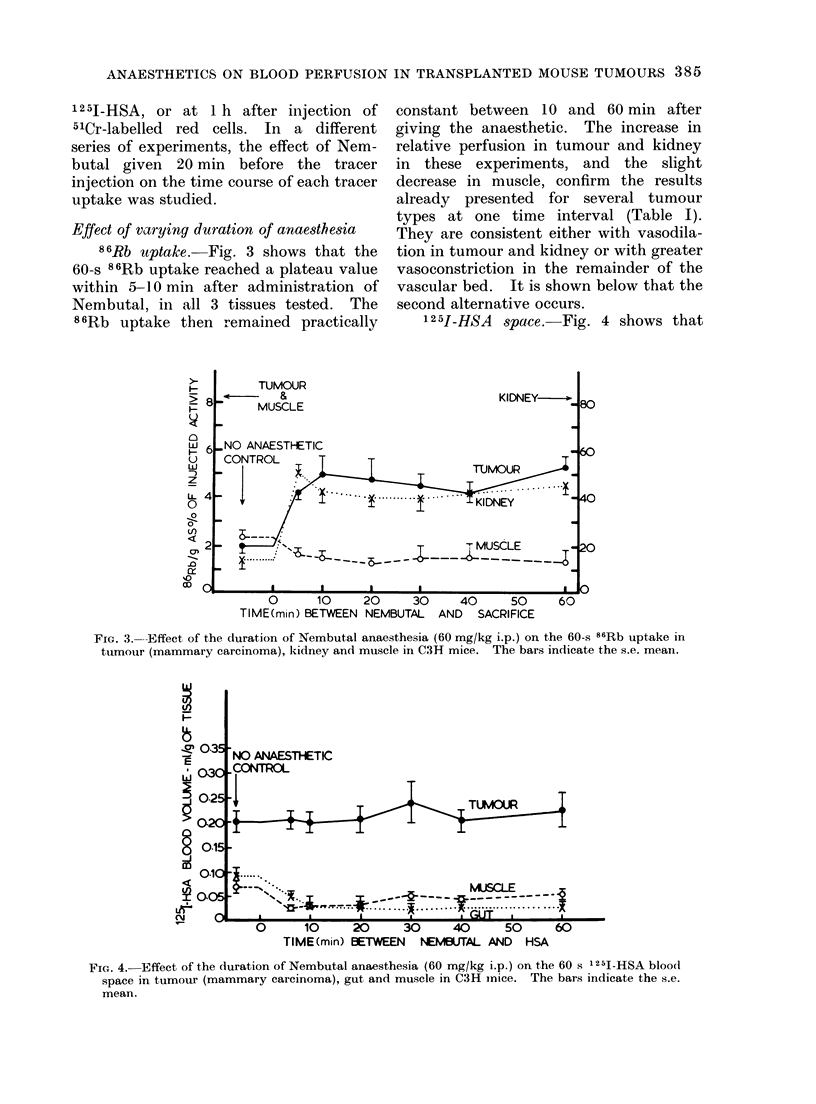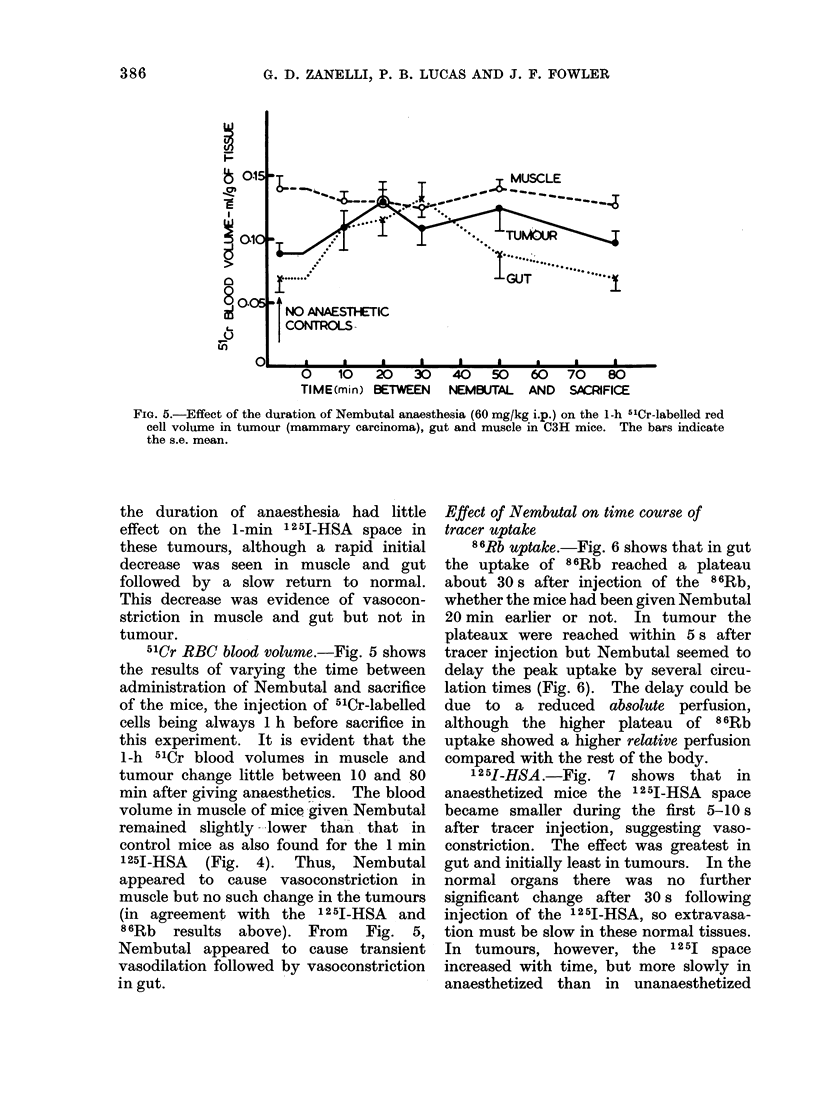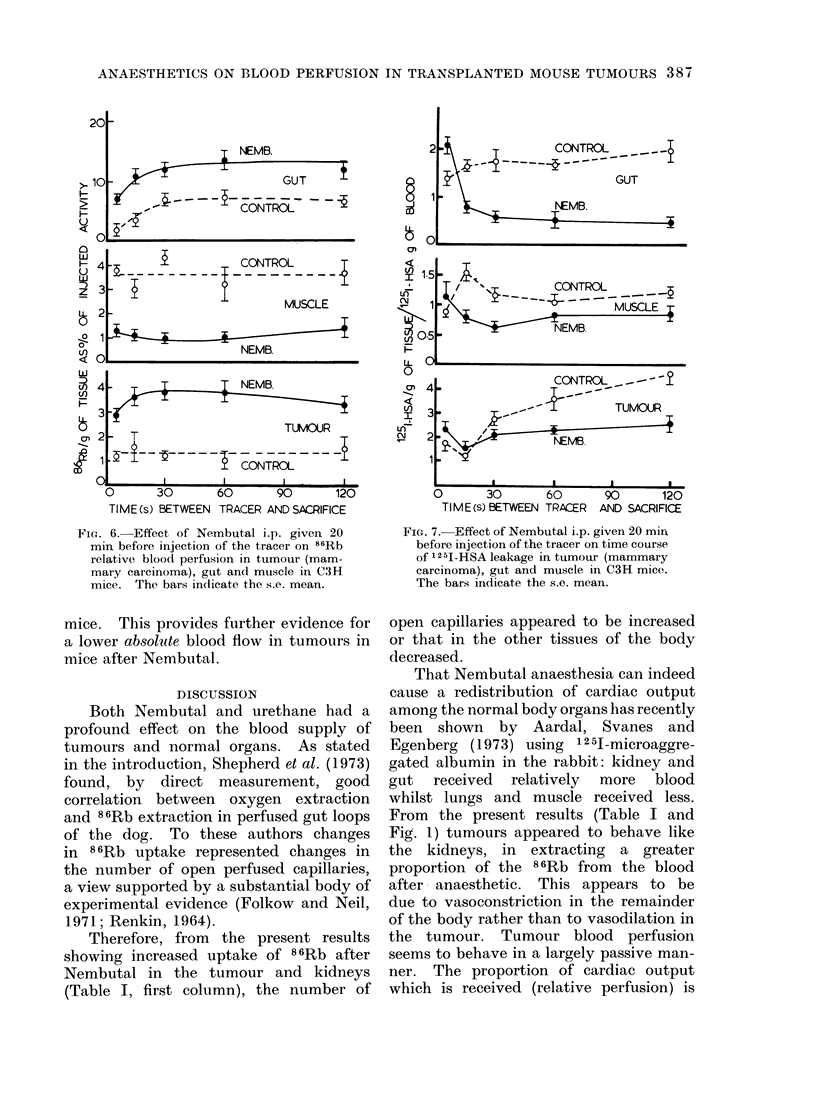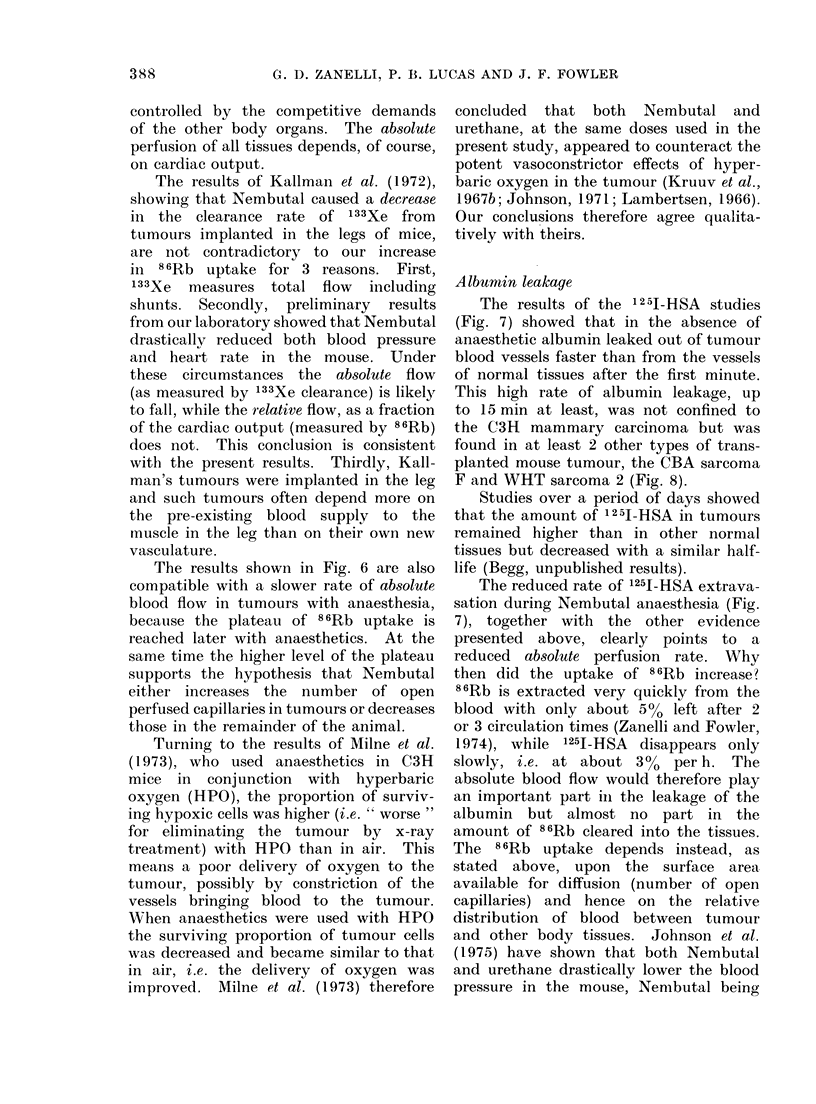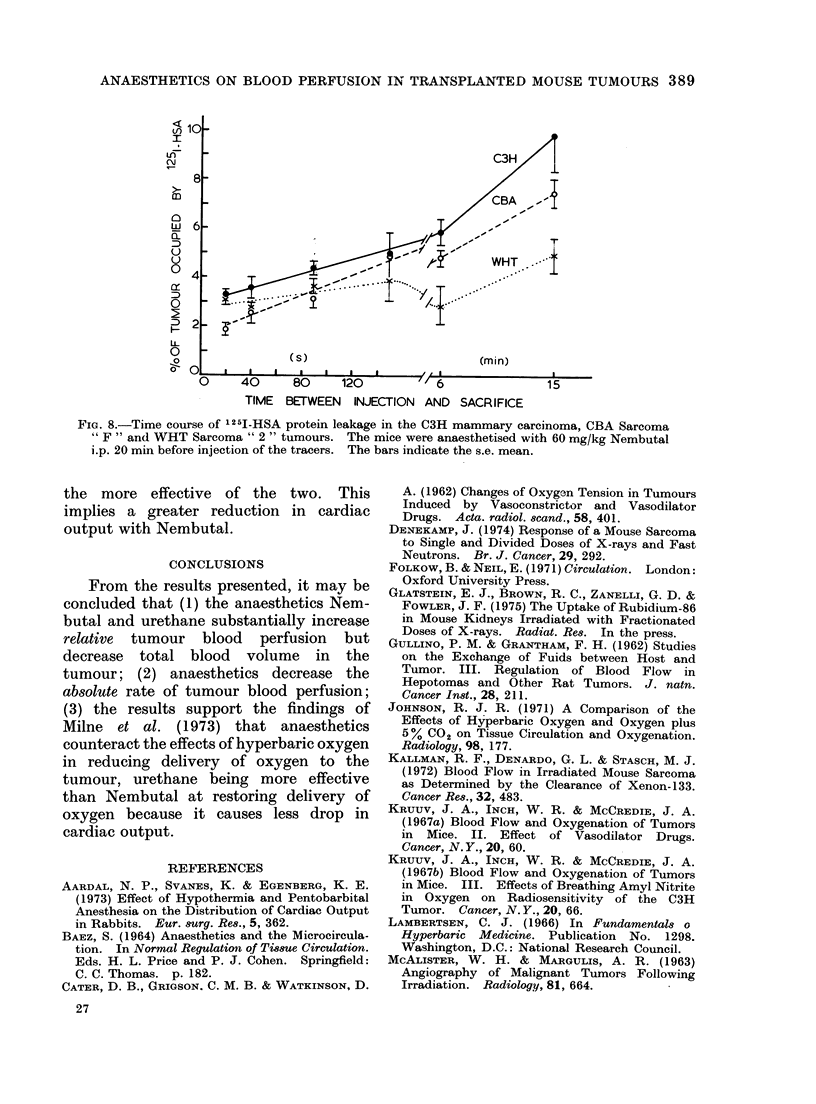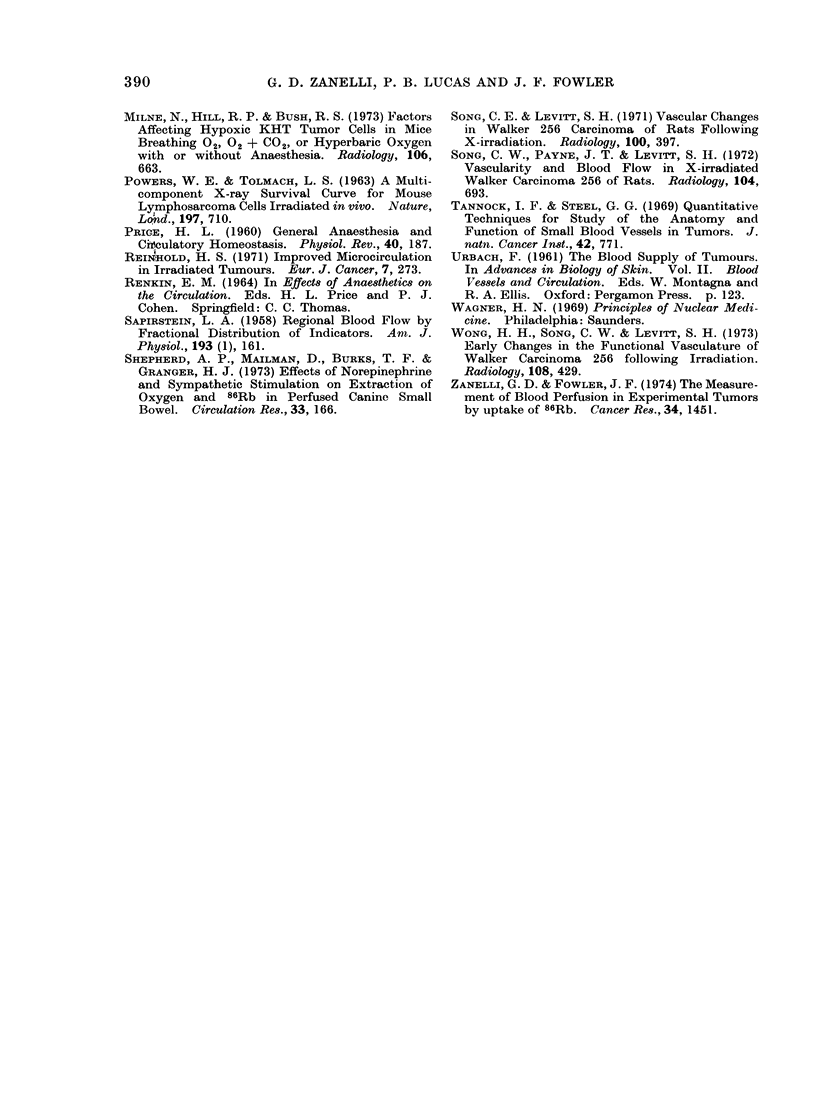# The effect of anaesthetics on blood perfusion in transplanted mouse tumours.

**DOI:** 10.1038/bjc.1975.238

**Published:** 1975-09

**Authors:** G. D. Zanelli, P. B. Lucas, J. F. Fowler

## Abstract

Rubidium-86, 125I-human serum albumin and 51Cr-labelled red cells have been used to investigate the effects of the anaesthetics Nembutal (pentobarbitone sodium) and urethane on blood perfusion, blood volume and albumin leakage in 5 types of transplanted mouse tumour and in normal organs. Nembutal was found to increase the relative blood perfusion by a factor of 1-3 to 2-0 in tumours and by a factor of 1-7 to 3-0 in kidneys but muscle perfusion fell to 0-3-0-5 that of controls. The effects of urethane were found to be dose dependent, generally in the same direction as for Nembutal, and smaller. Both anaesthetics reduced the blood volume of tumours (except for the C3H mammary carcinoma) and of kidneys by factors of 0-2 to 0-8. The duration of anaesthesia had no effect on the plateau values of relative blood perfusion and blood volume in either tumours or normal organs, but Nembutal delayed slightly the 86Rb uptake and decreased the rate of albumin leakage.


*Br. J. Cancer* (1975) **32**, 380

### THE EFFECT OF ANAESTHETICS ON BLOOD PERFUSION IN TRANSPLANTED MOUSE TUMOURS

G. D. ZANELLI, P. B. LUCAS AND J. F. FOWLER

*From the Gray Laboratory of the Cancer Research Campaign, Mount Vernon Hospital, Northwood, Middx. HA6 2RN*

Received 11 April 1975. Accepted 28 May 1975

**Summary.—Rubidium-86, $^{125}$I-human serum albumin and $^{51}$Cr-labelled red cells have been used to investigate the effects of the anaesthetics Nembutal (pentobarbitone sodium) and urethane on blood perfusion, blood volume and albumin leakage in 5 types of transplanted mouse tumour and in normal organs. Nembutal was found to increase the relative blood perfusion by a factor of 1·3 to 2·0 in tumours and by a factor of 1·7 to 3·0 in kidneys but muscle perfusion fell to 0·3–0·5 that of controls. The effects of urethane were found to be dose dependent, generally in the same direction as for Nembutal, and smaller. Both anaesthetics reduced the blood volume of tumours (except for the C3H mammary carcinoma) and of kidneys by factors of 0·2 to 0·8. The duration of anaesthesia had no effect on the plateau values of relative blood perfusion and blood volume in either tumours or normal organs, but Nembutal delayed slightly the $^{86}$Rb uptake and decreased the rate of albumin leakage.**

ANAESTHETICS, and in particular pentobarbitone sodium and urethane, have been used in most radiobiological studies on transplanted animal tumours and on normal tissues (McAlister and Margulis, 1963; Kruuv, Inch and McCredie, 1967; Denekamp, 1974), although some experimenters have avoided anaesthetics. All known anaesthetics have more or less pronounced effects on the microcirculation of the normal body organs, the magnitude of effect being dependent on the dose and the animal species (Price, 1960; Baez, 1964). The question whether or not the vasculature of malignant tumours reacts to vasoactive stimuli differently from the normal organ vasculature has received little experimental attention and the few reports on the subject reach conflicting conclusions (Urbach, 1961; Gullino and Grantham, 1962; Kruuv *et al.*, 1967*a*; Cater, Grigson and Watkinson, 1962; Powers and Tolmach, 1963). It is surprising therefore to find that even in reports of experiments designed to investigate the anatomy and function of tumour microcirculation (McAlister and Margulis, 1963; Tannock and Steel, 1969) or the effects of radiation upon microcirculation (Reinhold, 1971; Song and Levitt, 1971; Song, Payne and Levitt, 1972; Wong, Song and Levitt, 1973) anaesthetics should have been used without due account having been taken of their possible effects on the experimental results.

The first quantitative evidence of the effect of an anaesthetic on tumour blood supply seems to have appeared in a report by Kallman, Denardo and Stasch (1972), where it was shown that 60 mg/kg of Nembutal i.p. reduced the rate of blood flow through tumours (determined by $^{133}$Xe clearance) by a factor of nearly 2. These authors did not investigate the mechanism further. The most important (and probably the only) report directly concerned with the effects of anaesthetics on radiocurability of tumours is that of Milne, Hill and Bush (1973), where it was shown that Nembutal and urethane decreased the hypoxic fraction of tumour cells in mice breathing high pressure oxygen (HPO). The effect of the anaesthetics without HPO was not investigated although anaesthetics alone are of course commonly used.

We have used radioactive tracer methods to investigate the effects of the anaesthetics Nembutal and urethane on blood flow, protein leakage and blood volume in 5 transplanted mouse tumours and some normal organs in the same animals. One of the tumour systems (first generation transplanted mammary carcinomata in C3H mice) was then chosen for a more detailed study of the mode of action of the anaesthetics.

The use of $^{51}$Cr-labelled red blood cells and $^{125}$I-human serum albumin to study organ blood volume and protein leakage are standard methods (Folkow and Neil, 1971; Wagner, 1969). The $^{86}$Rb clearance method was established as a means for quantitating relative organ blood flow by Sapirstein (1958). More recent evidence shows that $^{86}$Rb can be used to obtain information on capillary permeability (P) and surface area (S), since these parameters are related by the expression $C = Q(1 - e^{-PS/Q})$, where C is the rate of clearance of a substance from the blood, Q is the blood flow and $e$ is the base of the natural logarithms (Folkow and Neil, 1971; Renkin, 1964). The product PS is a measure of the total number of capillaries open to circulation at any given time. Furthermore Shepherd *et al.* (1973) have shown, in the small bowel of the dog, that there was an excellent correlation between $^{86}$Rb extraction and uptake of oxygen by tissue, as assessed by the arteriovenous $O_2$ difference. Changes in oxygen uptake induced by infusion of noradrenaline or by sympathetic nerve stimulation were paralleled by changes in $^{86}$Rb uptake. This is an important consideration in relation to the proportion of cells which might be hypoxic in a tumour (Milne *et al.*, 1973).

### MATERIALS AND METHODS

*The tumour systems.*—We have investigated the following 5 transplanted murine tumours: (i) first generation transplants of spontaneous mammary carcinomata in C3H mice; (ii) carcinoma " NT " in CBA mice; (iii) sarcoma " 2 " in WHT mice; (iv) fast growing sarcoma " F " in CBA mice; (v) slow growing sarcoma " S " in CBA mice.

All tumours were transplanted into the recipient mice by subcutaneous implantation of pieces of tumour approximately 1 mm$^3$ in size over the rib cage under Nembutal anaesthesia (60 mg/kg i.p.). Most experiments were carried out when the tumours reached an average size of about 7–10 mm diameter. The mice were bred at the Gray Laboratory.

*Tracers and methods.*—Organ and tumour blood perfusion were studied by means of $^{86}$Rb uptake (rubidium chloride, 3–5 mCi/ml, Radiochemical Centre, Amersham) according to the method set out by Sapirstein (1958) and described in detail in a previous publication (Zanelli and Fowler, 1974). Briefly, approximately 2·5 $\mu$Ci of $^{86}$Rb in physiological saline were injected in 0·1 ml volumes into the tail vein of each animal. One minute later the animals were killed by decapitation and blood, tumour and other relevant organs (kidneys, gut, muscle) were collected, placed in double glass vials and counted for 200 s in an autogamma counter. The injection solution also contained approximately 0·25 $\mu$Ci/0·1 ml of iodinated human serum albumin ($^{125}$I-HSA, 50 $\mu$Ci/ml, Radiochemical Centre, Amsersham) to obtain an indication of blood volume and protein leakage, and was counted simultaneously with the $^{86}$Rb.

Tumour and organ blood volumes were also determined by means of $^{51}$Cr-labelled red blood cells (sodium-chromate, 1000 $\mu$Ci/ml, Radiochemical Centre, Amersham). For this purpose blood (5–7 ml) was collected from syngeneic mice into a centrifuge tube containing 1·0 ml of acid-citrate-dextrose solution and approximately 250 $\mu$Ci of $^{51}$Cr was added to it. After 30 min at room temperature, during which it was frequently gently shaken, the blood was centrifuged at 1000 rev/min for 15 min, the supernatant was discarded and replaced with sterile saline. This washing procedure was repeated 3 times and after the last washing the packed red cells were made up to the original volume of blood by adding saline. Exactly 0·1 ml of the labelled red cells was injected into the tail vein of each mouse. One h later the mice were killed by decapitation and blood, tumour and various organs were collected and counted. The 1 h time interval was used since we had shown that by this time all tumours had reached a plateau of radioactivity which lasted for several hours.

*Anaesthetics.*—The anaesthetics tested were pentobarbitone sodium (Nembutal, Abbott Laboratories, Queenborough, England) diluted in physiological saline and given in doses of 60 mg/kg body weight intraperitoneally, and urethane (Urethane *B.P.*, Koch-Light Laboratories, Colnbrook, England) dissolved in saline in doses of 1·0 g/kg body weight i.p. The volumes of anaesthetic injected were 0·1 ml/10 g body weight. In the first part of the investigation the anaesthetics were given 20 min ($\pm$3 min) before the injection of the radioactive tracers, *i.e.* 21 $\pm$ 3 min before sacrifice in the experiments with $^{86}$Rb + $^{125}$I-HSA or 81 $\pm$ 3 min before sacrifice in the $^{51}$Cr-labelled red cells experiments. It is shown below that the time interval between injection of anaesthetic and sacrifice gives no significant difference in the 1 min $^{86}$Rb uptake or $^{125}$I-HSA blood volume.

*Choice of normal organs for study.*—In the first part of the project, the kidney and muscle from the upper hind leg were used. The kidney was chosen because of previous experience in this laboratory (Glatstein *et al.*, 1975) with this organ and because of its large vascular supply, and the muscle because it shows dramatic changes in blood perfusion when subjected to vasoactive stimuli (Folkow and Neil, 1971). Later, the small bowel was used instead of the kidneys as a more sensitive monitor of visceral blood distribution and because it behaves much like the kidney under vasoactive stimuli.

### RESULTS

### $^{86}$*Rb uptake at* 1 *min* (*relative perfusion*)

The results of the first series of experiments are set out in Table I. The body of the table shows the *ratio* of the values obtained for anaesthetized and non-anaesthetized mice $\pm$ the standard error of the ratio. The latter was calculated as the square root of the sum of the squares of the *percent* standard errors of the 2 terms involved in each ratio and then converted into absolute standard errors. Student's *t* test, calculated as (Difference of Means)/(Standard Error of Difference), was applied to the ratios and those which are not significant at the 5% level are denoted by (NS). Nembutal always increased relative tumour perfusion, by a factor of 1·3 to 2·0, but severely reduced that of muscle (0·3 to 0·5). All 5 tumours seemed to behave like the kidneys. The increased $^{86}$Rb uptake in these organs suggested an increase in the total surface area of the exchange vessels relative to all other organs combined, *i.e.* a greater number of open capillaries in the tumour or fewer in the remainder of the animal. In this redistribution of fractional cardiac output some of the "extra" blood perfusing tumour and kidneys was presumably shunted away from the skeletal muscle as evidenced by reduced $^{86}$Rb uptake in muscle.

The effect of urethane on tumour perfusion was variable and seemed to depend on the depth of anaesthesia, *i.e.* on the dose. This is shown in Fig. 1. Low doses of urethane actually decreased tumour perfusion but higher doses increased it (Fig. 1). Muscle perfusion decreased with increasing dose, as with Nembutal. In summary, the kidneys and tumours responded to high doses of urethane much as they did to Nembutal. Muscle perfusion, although generally depressed by both agents, was less affected by urethane than by Nembutal.

### $^{125}$*I-HSA at* 1 *min* (*blood volume*) *and* $^{51}$*Cr at* 1 *h* (*blood volume*)

The 1 min $^{125}$I-HSA tumour "blood volumes" were usually about 1·5 times greater than those obtained using $^{51}$Cr administered any time between 10 and 60 min, so presumably very rapid leakage of the HSA takes place. This ratio rose from 1·5 to 3 between 1 and 15 min (Fig. 2), *i.e.* the rate of leakage decreased with time. This pattern is similar in gut and muscle.

However, in the proportional change caused by the agents under investigation,

TABLE I.—*Effect of Nembutal* (60 *mg/kg i.p.*) *and Urethane* (1 *g/kg i.p.*) *on Blood Perfusion and Blood Volume in Tumours and Normal Organs. Ratios of Anaesthetized/Unaesthetized Mice*

| | $^{86}$Rb/g at 1 min (no. of mice) | | | $^{125}$I-HSA blood volume at 1 min | | | $^{51}$Cr-labelled red cells blood volume at 1 h | | |
|---|---|---|---|---|---|---|---|---|---|
| | Tumour | Kidney | Muscle | Tumour | Kidney | Muscle | Tumour | Kidney | Muscle |
| C3H mammary carcinoma | | | | | | | | | |
| Nembutal | 1·7±0·4* (21)† | 2·9±0·6 | 0·5±0·03 | 0·8±0·1 | 0·5±0·1 | 0·5±0·04 | 2·2±0·7 (30) | 0·8±0·2 (NS) | 0·9±0·2 (NS) |
| Urethane | 1·5±0·3 (19) | 1·7±0·4 | 0·6±0·1 | 1·2±0·25 (NS) | 0·7±0·15 (NS) | 0·9±0·07 (NS) | 0·5±0·03 (14) | 1·7±0·3 | 1·2±0·25 (NS) |
| CBA " NT " carc. | | | | | | | | | |
| Nembutal | 1·8±0·6 (36) | 2·8±0·9 | 0·3±0·6 | 0·7±0·2 (NS) | 0·2±0·01 | 0·7±0·15 | 0·6±0·1 (16) | 0·9±0·1 (NS) | 0·9±0·25 (NS) |
| Urethane | 1·6±0·5 (12) | 2·4±0·6 | 0·9±0·2 (NS) | 0·6±0·1 | 0·4±0·02 | 0·2±0·05 | 0·6±0·15 (16) | 1·4±0·3 (NS) | 1·1±0·2 (NS) |
| CBA " F " sarc. | | | | | | | | | |
| Nembutal | 1·3±0·5 (36) | 3·1±0·5 | 0·3±0·04 | 0·5±0·1 | 0·3±0·01 | 0·9±0·1 (NS) | 0·5±0·08 (13) | 0·5±0·06 | 0·7±0·09 |
| Urethane | 1·6±0·5 (39) | 2·7±0·7 | 0·8±0·1 (NS) | 0·6±0·08 | 0·5±0·05 | 1·0±0·25 (NS) | 0·7±0·09 (11) | 1·1±0·15 (NS) | 1·0±0·2 (NS) |
| CBA " S " sarc. | | | | | | | | | |
| Nembutal | 1·9±0·7 (19) | 2·8±0·3 | 0·3±0·03 | 1·0±0·2 (NS) | 0·4±0·03 | 0·8±0·15 | 0·8±0·2 (14) (NS) | 0·5±0·05 | 1·0±0·2 (NS) |
| Urethane | 0·9±0·1 (14) (NS) | 2·3±0·6 | 0·9±0·09 | 0·2±0·06 | 0·5±0·03 | 0·4±0·07 | 0·9±0·25 (14) (NS) | 0·9±0·25 (NS) | 0·8±0·1 (NS) |
| WHT " 2 " sarc. | | | | | | | | | |
| Nembutal | 1·5±0·2 (27) | 1·7±0·2 | 0·4±0·1 | 0·7±0·2 | 0·7±0·04 | 0·9±0·17 (NS) | 0·5±0·1 (14) | 0·6±0·1 | 1·1±0·25 |
| Urethane | 0·9±0·3 (11) (NS) | 1·5±0·4 | 0·6±0·1 | 0·4±0·04 | 0·7±0·06 | 0·9±0·1 (NS) | 0·4±0·08 (11) | 1·2±0·2 (NS) | 1·8±0·4 |

* S.e. mean for the ratio.

† Number of mice used in this set of determinations for tumour, kidney and muscle.

(NS) = not significant at the 5% level.

FIG. 1.—Effect of the dose of urethane i.p. on the 60-s $^{86}$Rb uptake in tumour (mammary carcinoma), kidney and muscle in C3H mice. The bars indicate the s.e. mean of the ratio.

FIG. 2.—$^{125}$I-HSA " blood space " as a function of time between injection of the HSA and sacrifice. Also shown is the 1-h $^{51}$Cr-labelled red cell space measured in the same mice. No anaesthetic. The bars indicate the s.e. mean.

there was good agreement between the 1 min $^{125}$I-HSA and the 1 h $^{51}$Cr blood volume in 7 out of 10 of the tumour measurements in Table I.

Nembutal either decreased or left unaffected the $^{125}$I-HSA or $^{51}$Cr content, in tumours, but greatly decreased that of the kidney and somewhat reduced that of muscle. In muscle this change was more marked with $^{125}$I-HSA than with $^{51}$Cr. This result is consistent with greater vasoconstriction in the normal tissues than in the tumour.

Urethane also generally decreased the tumour $^{125}$I-HSA or $^{51}$Cr content with only the C3H mammary carcinoma $^{125}$I-HSA value remaining practically unaffected. $^{125}$I-HSA in kidney and muscle was generally decreased by similar amounts, but $^{51}$Cr content was not.

In the second part of the project, a single type of tumour was used to investigate the effects of varying the time between injection of Nembutal and injection of the tracers, *i.e.* the duration of anaesthesia. The animals were killed as usual at 1 min after injection of $^{86}$Rb +

$^{125}$I-HSA, or at 1 h after injection of $^{51}$Cr-labelled red cells. In a different series of experiments, the effect of Nembutal given 20 min before the tracer injection on the time course of each tracer uptake was studied.

*Effect of varying duration of anaesthesia*

*$^{86}$Rb uptake.*—Fig. 3 shows that the 60-s $^{86}$Rb uptake reached a plateau value within 5–10 min after administration of Nembutal, in all 3 tissues tested. The $^{86}$Rb uptake then remained practically constant between 10 and 60 min after giving the anaesthetic. The increase in relative perfusion in tumour and kidney in these experiments, and the slight decrease in muscle, confirm the results already presented for several tumour types at one time interval (Table I). They are consistent either with vasodilation in tumour and kidney or with greater vasoconstriction in the remainder of the vascular bed. It is shown below that the second alternative occurs.

*$^{125}$I-HSA space.*—Fig. 4 shows that

FIG. 3.—Effect of the duration of Nembutal anaesthesia (60 mg/kg i.p.) on the 60-s $^{86}$Rb uptake in tumour (mammary carcinoma), kidney and muscle in C3H mice. The bars indicate the s.e. mean.

FIG. 4.—Effect of the duration of Nembutal anaesthesia (60 mg/kg i.p.) on the 60 s $^{125}$I-HSA blood space in tumour (mammary carcinoma), gut and muscle in C3H mice. The bars indicate the s.e. mean.

FIG. 5.—Effect of the duration of Nembutal anaesthesia (60 mg/kg i.p.) on the 1-h $^{51}$Cr-labelled red cell volume in tumour (mammary carcinoma), gut and muscle in C3H mice. The bars indicate the s.e. mean.

the duration of anaesthesia had little effect on the 1-min $^{125}$I-HSA space in these tumours, although a rapid initial decrease was seen in muscle and gut followed by a slow return to normal. This decrease was evidence of vasoconstriction in muscle and gut but not in tumour.

*$^{51}$Cr RBC blood volume.*—Fig. 5 shows the results of varying the time between administration of Nembutal and sacrifice of the mice, the injection of $^{51}$Cr-labelled cells being always 1 h before sacrifice in this experiment. It is evident that the 1-h $^{51}$Cr blood volumes in muscle and tumour change little between 10 and 80 min after giving anaesthetics. The blood volume in muscle of mice given Nembutal remained slightly lower than that in control mice as also found for the 1 min $^{125}$I-HSA (Fig. 4). Thus, Nembutal appeared to cause vasoconstriction in muscle but no such change in the tumours (in agreement with the $^{125}$I-HSA and $^{86}$Rb results above). From Fig. 5, Nembutal appeared to cause transient vasodilation followed by vasoconstriction in gut.

*Effect of Nembutal on time course of tracer uptake*

*$^{86}$Rb uptake.*—Fig. 6 shows that in gut the uptake of $^{86}$Rb reached a plateau about 30 s after injection of the $^{86}$Rb, whether the mice had been given Nembutal 20 min earlier or not. In tumour the plateaux were reached within 5 s after tracer injection but Nembutal seemed to delay the peak uptake by several circulation times (Fig. 6). The delay could be due to a reduced *absolute* perfusion, although the higher plateau of $^{86}$Rb uptake showed a higher *relative* perfusion compared with the rest of the body.

*$^{125}$I-HSA.*—Fig. 7 shows that in anaesthetized mice the $^{125}$I-HSA space became smaller during the first 5–10 s after tracer injection, suggesting vasoconstriction. The effect was greatest in gut and initially least in tumours. In the normal organs there was no further significant change after 30 s following injection of the $^{125}$I-HSA, so extravasation must be slow in these normal tissues. In tumours, however, the $^{125}$I space increased with time, but more slowly in anaesthetized than in unanaesthetized

FIG. 6.—Effect of Nembutal i.p. given 20 min before injection of the tracer on $^{86}$Rb relative blood perfusion in tumour (mammary carcinoma), gut and muscle in C3H mice. The bars indicate the s.e. mean.

FIG. 7.—Effect of Nembutal i.p. given 20 min before injection of the tracer on time course of $^{125}$I-HSA leakage in tumour (mammary carcinoma), gut and muscle in C3H mice. The bars indicate the s.e. mean.

mice. This provides further evidence for a lower *absolute* blood flow in tumours in mice after Nembutal.

### DISCUSSION

Both Nembutal and urethane had a profound effect on the blood supply of tumours and normal organs. As stated in the introduction, Shepherd *et al.* (1973) found, by direct measurement, good correlation between oxygen extraction and $^{86}$Rb extraction in perfused gut loops of the dog. To these authors changes in $^{86}$Rb uptake represented changes in the number of open perfused capillaries, a view supported by a substantial body of experimental evidence (Folkow and Neil, 1971; Renkin, 1964).

Therefore, from the present results showing increased uptake of $^{86}$Rb after Nembutal in the tumour and kidneys (Table I, first column), the number of open capillaries appeared to be increased or that in the other tissues of the body decreased.

That Nembutal anaesthesia can indeed cause a redistribution of cardiac output among the normal body organs has recently been shown by Aardal, Svanes and Egenberg (1973) using $^{125}$I-microaggregated albumin in the rabbit: kidney and gut received relatively more blood whilst lungs and muscle received less. From the present results (Table I and Fig. 1) tumours appeared to behave like the kidneys, in extracting a greater proportion of the $^{86}$Rb from the blood after anaesthetic. This appears to be due to vasoconstriction in the remainder of the body rather than to vasodilation in the tumour. Tumour blood perfusion seems to behave in a largely passive manner. The proportion of cardiac output which is received (relative perfusion) is controlled by the competitive demands of the other body organs. The *absolute* perfusion of all tissues depends, of course, on cardiac output.

The results of Kallman *et al.* (1972), showing that Nembutal caused a *decrease* in the clearance rate of $^{133}Xe$ from tumours implanted in the legs of mice, are not contradictory to our increase in $^{86}Rb$ uptake for 3 reasons. First, $^{133}Xe$ measures total flow including shunts. Secondly, preliminary results from our laboratory showed that Nembutal drastically reduced both blood pressure and heart rate in the mouse. Under these circumstances the *absolute* flow (as measured by $^{133}Xe$ clearance) is likely to fall, while the *relative* flow, as a fraction of the cardiac output (measured by $^{86}Rb$) does not. This conclusion is consistent with the present results. Thirdly, Kallman's tumours were implanted in the leg and such tumours often depend more on the pre-existing blood supply to the muscle in the leg than on their own new vasculature.

The results shown in Fig. 6 are also compatible with a slower rate of *absolute* blood flow in tumours with anaesthesia, because the plateau of $^{86}Rb$ uptake is reached later with anaesthetics. At the same time the higher level of the plateau supports the hypothesis that Nembutal either increases the number of open perfused capillaries in tumours or decreases those in the remainder of the animal.

Turning to the results of Milne *et al.* (1973), who used anaesthetics in C3H mice in conjunction with hyperbaric oxygen (HPO), the proportion of surviving hypoxic cells was higher (*i.e.* " worse " for eliminating the tumour by x-ray treatment) with HPO than in air. This means a poor delivery of oxygen to the tumour, possibly by constriction of the vessels bringing blood to the tumour. When anaesthetics were used with HPO the surviving proportion of tumour cells was decreased and became similar to that in air, *i.e.* the delivery of oxygen was improved. Milne *et al.* (1973) therefore concluded that both Nembutal and urethane, at the same doses used in the present study, appeared to counteract the potent vasoconstrictor effects of hyperbaric oxygen in the tumour (Kruuv *et al.*, 1967*b*; Johnson, 1971; Lambertsen, 1966). Our conclusions therefore agree qualitatively with theirs.

*Albumin leakage*

The results of the $^{125}I$-HSA studies (Fig. 7) showed that in the absence of anaesthetic albumin leaked out of tumour blood vessels faster than from the vessels of normal tissues after the first minute. This high rate of albumin leakage, up to 15 min at least, was not confined to the C3H mammary carcinoma but was found in at least 2 other types of transplanted mouse tumour, the CBA sarcoma F and WHT sarcoma 2 (Fig. 8).

Studies over a period of days showed that the amount of $^{125}I$-HSA in tumours remained higher than in other normal tissues but decreased with a similar half-life (Begg, unpublished results).

The reduced rate of $^{125}I$-HSA extravasation during Nembutal anaesthesia (Fig. 7), together with the other evidence presented above, clearly points to a reduced *absolute* perfusion rate. Why then did the uptake of $^{86}Rb$ increase? $^{86}Rb$ is extracted very quickly from the blood with only about 5% left after 2 or 3 circulation times (Zanelli and Fowler, 1974), while $^{125}I$-HSA disappears only slowly, *i.e.* at about 3% per h. The absolute blood flow would therefore play an important part in the leakage of the albumin but almost no part in the amount of $^{86}Rb$ cleared into the tissues. The $^{86}Rb$ uptake depends instead, as stated above, upon the surface area available for diffusion (number of open capillaries) and hence on the relative distribution of blood between tumour and other body tissues. Johnson *et al.* (1975) have shown that both Nembutal and urethane drastically lower the blood pressure in the mouse, Nembutal being

FIG. 8.—Time course of $^{125}$I-HSA protein leakage in the C3H mammary carcinoma, CBA Sarcoma " F " and WHT Sarcoma " 2 " tumours. The mice were anaesthetised with 60 mg/kg Nembutal i.p. 20 min before injection of the tracers. The bars indicate the s.e. mean.

the more effective of the two. This implies a greater reduction in cardiac output with Nembutal.

### CONCLUSIONS

From the results presented, it may be concluded that (1) the anaesthetics Nembutal and urethane substantially increase *relative* tumour blood perfusion but decrease total blood volume in the tumour; (2) anaesthetics decrease the *absolute* rate of tumour blood perfusion; (3) the results support the findings of Milne *et al.* (1973) that anaesthetics counteract the effects of hyperbaric oxygen in reducing delivery of oxygen to the tumour, urethane being more effective than Nembutal at restoring delivery of oxygen because it causes less drop in cardiac output.

### REFERENCES

AARDAL, N. P., SVANES, K. & EGENBERG, K. E. (1973) Effect of Hypothermia and Pentobarbital Anesthesia on the Distribution of Cardiac Output in Rabbits. *Eur. surg. Res.*, **5**, 362.

BAEZ, S. (1964) Anaesthetics and the Microcirculation. In *Normal Regulation of Tissue Circulation*. Eds. H. L. Price and P. J. Cohen. Springfield: C. C. Thomas. p. 182.

CATER, D. B., GRIGSON, C. M. B. & WATKINSON, D. A. (1962) Changes of Oxygen Tension in Tumours Induced by Vasoconstrictor and Vasodilator Drugs. *Acta. radiol. scand.*, **58**, 401.

DENEKAMP, J. (1974) Response of a Mouse Sarcoma to Single and Divided Doses of X-rays and Fast Neutrons. *Br. J. Cancer*, **29**, 292.

FOLKOW, B. & NEIL, E. (1971) *Circulation*. London: Oxford University Press.

GLATSTEIN, E. J., BROWN, R. C., ZANELLI, G. D. & FOWLER, J. F. (1975) The Uptake of Rubidium-86 in Mouse Kidneys Irradiated with Fractionated Doses of X-rays. *Radiat. Res.* In the press.

GULLINO, P. M. & GRANTHAM, F. H. (1962) Studies on the Exchange of Fuids between Host and Tumor. III. Regulation of Blood Flow in Hepotomas and Other Rat Tumors. *J. natn. Cancer Inst.*, **28**, 211.

JOHNSON, R. J. R. (1971) A Comparison of the Effects of Hyperbaric Oxygen and Oxygen plus 5% $CO_2$ on Tissue Circulation and Oxygenation. *Radiology*, **98**, 177.

KALLMAN, R. F., DENARDO, G. L. & STASCH, M. J. (1972) Blood Flow in Irradiated Mouse Sarcoma as Determined by the Clearance of Xenon-133. *Cancer Res.*, **32**, 483.

KRUUV, J. A., INCH, W. R. & MCCREDIE, J. A. (1967*a*) Blood Flow and Oxygenation of Tumors in Mice. II. Effect of Vasodilator Drugs. *Cancer, N.Y.*, **20**, 60.

KRUUV, J. A., INCH, W. R. & MCCREDIE, J. A. (1967*b*) Blood Flow and Oxygenation of Tumors in Mice. III. Effects of Breathing Amyl Nitrite in Oxygen on Radiosensitivity of the C3H Tumor. *Cancer, N.Y.*, **20**, 66.

LAMBERTSEN, C. J. (1966) In *Fundamentals o Hyperbaric Medicine*. Publication No. 1298. Washington, D.C.: National Research Council.

MCALISTER, W. H. & MARGULIS, A. R. (1963) Angiography of Malignant Tumors Following Irradiation. *Radiology*, **81**, 664.

MILNE, N., HILL, R. P. & BUSH, R. S. (1973) Factors Affecting Hypoxic KHT Tumor Cells in Mice Breathing $O_2$, $O_2 + CO_2$, or Hyperbaric Oxygen with or without Anaesthesia. *Radiology*, **106**, 663.

POWERS, W. E. & TOLMACH, L. S. (1963) A Multi-component X-ray Survival Curve for Mouse Lymphosarcoma Cells Irradiated *in vivo*. *Nature, Lond.*, **197**, 710.

PRICE, H. L. (1960) General Anaesthesia and Circulatory Homeostasis. *Physiol. Rev.*, **40**, 187.

REINHOLD, H. S. (1971) Improved Microcirculation in Irradiated Tumours. *Eur. J. Cancer*, **7**, 273.

RENKIN, E. M. (1964) In *Effects of Anaesthetics on the Circulation*. Eds. H. L. Price and P. J. Cohen. Springfield: C. C. Thomas.

SAPIRSTEIN, L. A. (1958) Regional Blood Flow by Fractional Distribution of Indicators. *Am. J. Physiol.*, **193** (1), 161.

SHEPHERD, A. P., MAILMAN, D., BURKS, T. F. & GRANGER, H. J. (1973) Effects of Norepinephrine and Sympathetic Stimulation on Extraction of Oxygen and $^{86}$Rb in Perfused Canine Small Bowel. *Circulation Res.*, **33**, 166.

SONG, C. E. & LEVITT, S. H. (1971) Vascular Changes in Walker 256 Carcinoma of Rats Following X-irradiation. *Radiology*, **100**, 397.

SONG, C. W., PAYNE, J. T. & LEVITT, S. H. (1972) Vascularity and Blood Flow in X-irradiated Walker Carcinoma 256 of Rats. *Radiology*, **104**, 693.

TANNOCK, I. F. & STEEL, G. G. (1969) Quantitative Techniques for Study of the Anatomy and Function of Small Blood Vessels in Tumors. *J. natn. Cancer Inst.*, **42**, 771.

URBACH, F. (1961) The Blood Supply of Tumours. In *Advances in Biology of Skin*. Vol. II. *Blood Vessels and Circulation*. Eds. W. Montagna and R. A. Ellis. Oxford: Pergamon Press. p. 123.

WAGNER, H. N. (1969) *Principles of Nuclear Medicine*. Philadelphia: Saunders.

WONG, H. H., SONG, C. W. & LEVITT, S. H. (1973) Early Changes in the Functional Vasculature of Walker Carcinoma 256 following Irradiation. *Radiology*, **108**, 429.

ZANELLI, G. D. & FOWLER, J. F. (1974) The Measurement of Blood Perfusion in Experimental Tumors by uptake of $^{86}$Rb. *Cancer Res.*, **34**, 1451.